# Design and Analysis of a Hybrid Annular Radial Magnetorheological Damper for Semi-Active In-Wheel Motor Suspension

**DOI:** 10.3390/s22103689

**Published:** 2022-05-12

**Authors:** Olivier Munyaneza, Pacifique Turabimana, Jong-Seok Oh, Seung-Bok Choi, Jung Woo Sohn

**Affiliations:** 1 Department of Aeronautics, Mechanical and Electronic Convergence Engineering, Graduate School, Kumoh National Institute of Technology, Gumi 39177, Gyeongbuk, Korea; olivismnza@gmail.com (O.M.); turabimanapacifique@gmail.com (P.T.); 2 Department of Future Automotive Engineering, Kongju National University, Cheonan 31080, Chungnam, Korea; jongseok@kongju.ac.kr; 3 Department of Mechanical Engineering, The State University of New York, Korea (SUNY Korea), Incheon 21985, Korea; 4 Department of Mechanical Engineering, Industrial University of Ho Chi Minh City (IUH), Ho Chi Minh City 70000, Vietnam; 5 Department of Mechanical Design Engineering, Kumoh National Institute of Technology, Gumi 39177, Gyeongbuk, Korea

**Keywords:** magnetorheological fluid, annular radial ducts, electric vehicle, in-wheel motor, permanent magnet

## Abstract

In this study, a novel hybrid annular radial magnetorheological damper (HARMRD) is proposed to improve the ride comfort of an electric vehicle (EV) powered by an in-wheel motor (IWM). The model primarily comprises annular-radial ducts in series with permanent magnets. Mathematical models representing the governing motions are formulated, followed by finite element analysis of the HARMRD to investigate the distribution of the magnetic field density and intensity of the magnetorheological (MR) fluid in both the annular and radial ducts. The optimized model generates a damping force of 87.3–445.7 N at the off-state (zero input current) with the excitation velocity ranging between 0 and 0.25 m/s. By contrast, the generated damping force varies from 3386.4 N to 3753.9 N at an input current of 1.5 A with the same velocity range as the off state. The damping forces obtained using the proposed model are 31.4% and 19.2% higher for the off-field and on-field states, respectively, compared with those of the conventional annular radial MR damper. The efficiency of the proposed model is evaluated by adopting two different vehicles: a conventional vehicle powered by an engine and an EV powered by an IWM. The simulation results demonstrate that the proposed HARMRD along with the skyhook controller significantly improves both the ride comfort and road-holding capability for both types of vehicles.

## 1. Introduction

Over the past two decades, vehicle comfort and road holding have become important considerations for car manufacturers owing to their ability to mitigate the effects caused by road imperfections. Semi-active suspensions have received considerable attention in this context. Semi-active devices use smart materials, such as magnetorheological (MR) fluids, which can shift from Newtonian to Bingham behavior within a microsecond under the influence of an external magnetic field. MR fluids offer a high yield strength and a wide range of operating temperatures [1,2]. MR dampers with MR fluids have advantages such as rapid response, low-voltage required to control the damping force, and structural simplicity [3,4,5]. Different structures of MR dampers have been studied and applied in control systems. For instance, Solomon et al. [6] designed a mono-tube MR damper by focusing on the performance of indices such as damping force, dynamic range, and valve ratio. They concluded that the design of an MR damper is highly dependent on the performance indices. The mono-tube-type MR damper is more commonly used than other models. However, it requires a larger volume to generate a high damping force, which is inconvenient for many applications [7,8]. Studies have attempted to enhance the damping force using the twin-tube model with low volume; however, this has proved difficulties owing to its complexity, hydraulic imbalance, and limited dynamic range [3,9]. Generating a highly controllable damping force with a compact model remains challenging. Jiang et al. [10] presented a novel design of a multichannel bypass MR damper operating under three working modes. Compared with conventional MR models, their model offers a larger adjustment range and improves the vibration response. However, this model is complex, requires a larger volume, and exhibits a high friction force, which can decay its damping force. By contrast, hybrid models generate a high damping force and address the problem of failure safety for field-off [11,12,13]. To achieve model simplicity and compactness while improving its damping characteristics, Bai et al. [14] proposed an annular-radial-duct MR damper to improve the efficiency of the MR damper. Their experimental results were compared with those obtained using an MR damper manufactured by LORD Corp. (Cary, NC, USA) (commercially available on the market), and the proposed model provided both a larger damping force and damping range compared with the commercial damper. Several studies have reported that the annular-radial model improves the controllable damping force and has a wide adjustable range [15,16,17]. However, research on the application of MR dampers based on the annular-radial model to conventional engine-powered or electric vehicles remains limited.

The main technical contribution of this study is the introduction of a novel type of MR damper to improve ride comfort designed based on the annular-radial model and its application to electric vehicles (EVs) powered by in-wheel motors (IWMs). Currently, several manufacturers are attempting to adopt this promising future technology in EVs. The vehicles powered by IWMs offer many benefits compared with internal combustion engine vehicles, such as high torque and vehicle efficiency up to 98% [18]. However, this technology has some serious limitations that can affect its reliability, such as increased sprung mass, which can deteriorate the dynamic performance of the vehicle, braking problems, and deterioration of motor bearing [19,20,21]. To address the challenges faced by annular-radial models that lack failure safety, we proposed a hybrid annular radial-type MR damper that achieves failure safety using a permanent magnet in addition to the annular and radial ducts that are connected in series. The model was designed and mathematically formulated to represent the governing motion. The performance of the new model was then analytically validated, and the results were compared with those reported in [14] to demonstrate the advantages of the proposed hybrid annular radial magnetorheological damper (HARMRD).

## 2. Configuration of HARMRD

**Figure 1:**
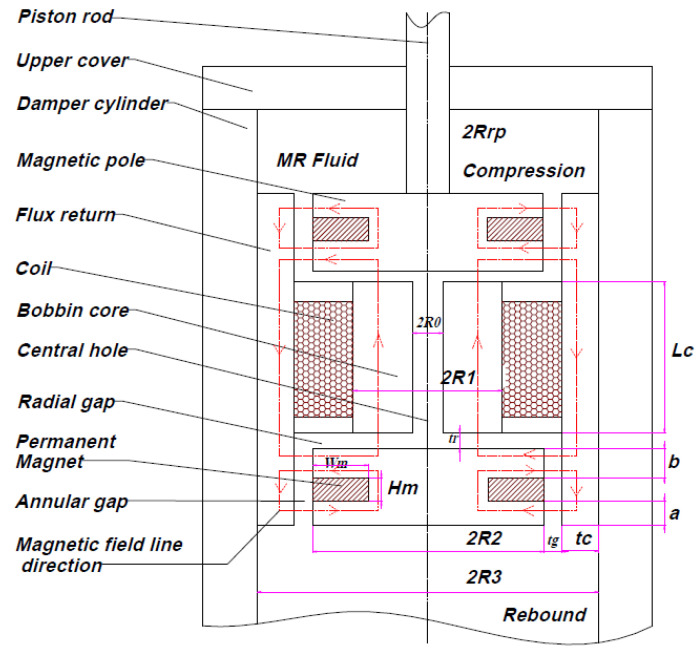
Schematic diagram of the proposed HARMRD.

The structural principle of the proposed HARMRD model is depicted in Figure 1. This figure presents a 2D schematic drawing of the HARMRD. The proposed model comprises a piston rod, permanent magnets (PMs), bobbin core, electromagnetic coil, two magnetic poles around the PM, magnetic core cylinder (flux return), damper cylinder, and other non-magnetic components such as washers and pins. Pins are used to connect the circular disc or poles to the bobbin core and ensure that a constant radial gap is formed. The annular gap is located between the outer core and the PM circumference and the inner circumference of the flux return. A permanent ring magnet is installed between the two poles at the top and bottom of the valve model. The material properties and dimensions are listed in Table 1.

The initial magnetic field is supplied by the PMs, wherein the field generated from the bobbin core passes through the annular gap (lower or upper), continues to the magnetic core cylinder (flux return), and returns to the core to complete a magnetic circuit cycle. The field generated by the PM has a greater influence on the annular channel than on the radial channel. To enhance the magnetic field generated by the magnets, a positive/negative current is applied to the coil to generate the primary magnetic field. Unlike the field generated by the magnets, that from the coil can be gradually increased by varying the current input. Depending on the position of the PM poles, the magnetic field of the coil can cancel or strengthen the field induced by PM. The PM field from the magnets creates yield stress in the MR fluid around the annular channel, which prevents the model from acting as a passive damper in the case of electric failure. As is evident from Figure 1, as the piston is compressed, the MR fluid flows from the upper annular gap into the upper radial gap. The fluid from the upper radial gap passes through the central hole of the core to the lower radial and annular gaps. This process is repeated during the rebound process. In addition, according to [22] the MR fluid fills the space between the channels because of the pressure drop, which is controlled by the magnetic field of the PM and electromagnetic coil.

## 3. Modeling of Hybrid Annular Radial MR Damper

The proposed model operates based on the principles of valve mode [8,23,24]. As shown in Figure 1, the model comprises two annular gaps, two radial gaps, and one central hole to facilitate the flow of the MR fluid from the low-pressure to the high-pressure side, and vice versa. The total pressure drop inside the valve is expressed as follows:

(1) $$\Delta_{p}=2\left(\Delta_{pa}+\Delta_{pr}\right)+\Delta_{p0},$$

where $\Delta_{pa}$, $\Delta_{pr},$ $\Delta_{p0}$ are the pressure drops in the annular gap, radial gap, and central hole, respectively. The pressure in the annular flow gap is expressed as [17]

(2) $$\Delta_{pa}=\Delta p_{\eta a}+\Delta p_{\tau a},$$

where $\Delta p_{\eta a}$ and $\Delta p_{\tau a}$ are the viscous pressure drop and field-dependent pressure drop, respectively, and are expressed as

(3) $$\Delta p_{\eta a}=\frac{6\eta Q_{a}t_{f}}{\pi t_{g}{}^{3}\left(R_{2}+0.5t_{g}\right)},$$

(4) $$\Delta p_{\tau a}=\frac{C_{a}t_{e}\tau_{\gamma a}}{t_{g}}.$$

The expressions for $Q_{a}$, $t_{f}$, and $t_{e}$ are deduced from Figure 1.

(5) $$Q_{a}=V_{p}A_{p},$$

(6) $$t_{f}=a+b+H_{m},$$

(7) $$t_{e}=a+b,$$

where η is the viscosity of the MR fluid; $Q_{a}$ is the volumetric flow rate of the MR fluid through the annular gap; $V_{p}$ is the piston velocity; $A_{p}$ is the effective area of the piston valve; $t_{f}$ is the length of the annular gap; $t_{e}$ is the effective length of the annular gap; a and b are the effective pole lengths below and above the magnet, respectively; and $H_{m}$ is the height of the magnet. The pressure drop in the radial gap is determined as

(8) $$\Delta_{pr}=\Delta p_{\eta r}+\Delta p_{\tau r},$$

where $\Delta p_{\eta r}$ and $\Delta p_{\tau r}$ are the pressure drops due to the MR fluid flowing in the radial gap and the yield stress, respectively.

(9) $$\Delta p_{\eta r}=\frac{6\eta Q_{r}}{\pi t_{g}{}^{3}}\ln\left(\frac{R_{2}}{R_{0}}\right)$$

(10) $$\Delta p_{\tau r}=\frac{C_{r}\left(R_{2}-R_{0}\right)\tau_{\gamma r}}{t_{g}}$$

The pressure drop, $\Delta_{p0}$, in the central hole is defined as

(11) $$\Delta_{p0}=\frac{8\eta QL_{c}}{\pi R_{0}{}^{4}},$$

where $L_{c}$ denotes the length of the central hole. By substituting Equations (3), (4), (9), (10) and (11) into Equation (1), the pressure drop becomes

(12) $$\Delta_{p}=2\left[\frac{6\eta Q_{a}t_{f}}{\pi t_{g}{}^{3}\left(R_{2}+0.5t_{g}\right)}+\frac{6\eta Q_{r}}{\pi t_{g}{}^{3}}\ln\left(\frac{R_{2}}{R_{0}}\right)+\frac{C_{a}t_{e}\tau_{\gamma a}}{t_{g}}+\frac{C_{r}\left(R_{2}-R_{0}\right)\tau_{\gamma r}}{t_{g}}\right]+\frac{8\eta QL_{c}}{\pi R_{0}{}^{4}}.$$

The viscous damper coefficient can be calculated from Equation (12) as follow

(13) $$C_{vic}=\left(2\left[\frac{6\eta t_{f}}{\pi t_{g}{}^{3}\left(R_{2}+0.5t_{g}\right)}+\frac{6\eta }{\pi t_{g}{}^{3}}\ln\left(\frac{R_{2}}{R_{0}}\right)\right]+\frac{8\eta QL_{c}}{\pi R_{0}{}^{4}}\right)\times A_{p}{}^{2}$$

The total damping force can be calculated using Equation (12) as

(14) $$F_{D}=2A_{p}\left\{\frac{6\eta Q_{a}t_{f}}{\pi t_{g}{}^{3}\left(R_{2}+0.5t_{g}\right)}+\frac{6\eta Q_{r}}{\pi t_{g}{}^{3}}\ln\left(\frac{R_{2}}{R_{0}}\right)+\frac{C_{a}t_{e}\tau_{\gamma a}}{t_{g}}+\frac{C_{r}\left(R_{2}-R_{0}\right)\tau_{\gamma r}}{t_{g}}\right\}+\frac{8\eta QL_{c}}{\pi R_{0}{}^{4}}A_{p}.$$

To evaluate the performance of the HARMRD, the dynamic range (*K*) was calculated as the ratio of the field-dependent force to the viscous damping force.

(15) $$K=\frac{F_{\tau }}{F_{\eta }}+1$$

## 4. Finite Element Analysis of HARMRD

**Figure 2:**
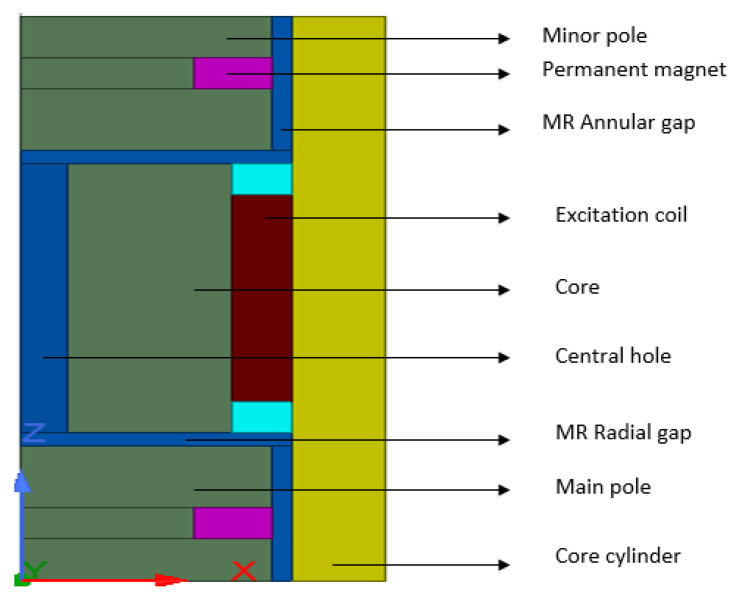
Axisymmetric model of the HARMRD.

**Figure 3:**
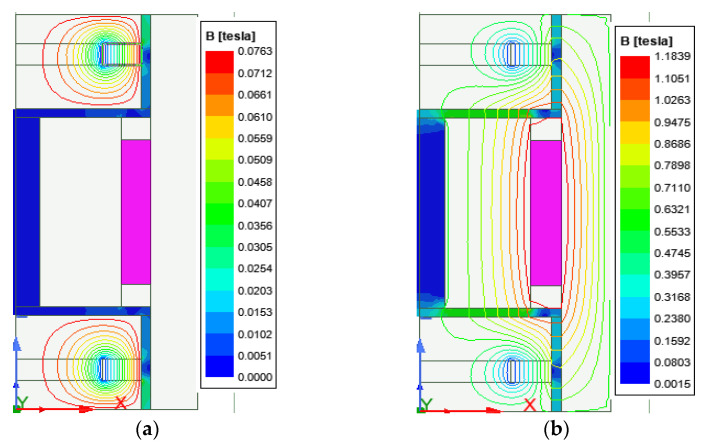
Distribution of magnetic field lines and flux density when: (**a**) only the PM is operating; (**b**) both the PM and coil are operating.

Mathematically, calculating the damping force and dynamic range of the HARMRD is challenging owing to the nonlinearity of the materials that make up the model. For convenience, finite element analysis (FEA) was performed using the ANSYS/Maxwell software. Figure 2 shows the axisymmetric model of the HARMR valve used in the simulation. The results of the two cases considered in the simulation are presented in Figure 3a,b. The distribution of the magnetic flux lines and magnetic flux density for the HARMR valve in the absence of excitation current to the coil is shown in Figure 3a. Evidently, the magnetic flux lines and density are concentrated around the PMs, and in this case, the proposed model continuously experiences a magnetic field around the annular channel. In addition, the magnetic fields generated in the annular gap and radial gap are 0.06 T and 0.01 T, respectively. Figure 3b shows the generated magnetic field lines and flux density when both the magnets and the electrical coil are operating. In this case, the initial magnetic field from the magnets is strengthened by the field generated by the coil after excitation. A current variation of 0–1.5 A with an interval of 0.25 A is supplied to the coil.

**Figure 4:**
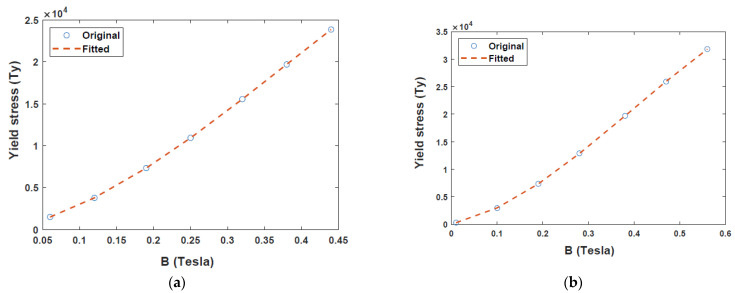
Polynomial curve fitting for variation of yield stress with magnetic flux density of MRF-132DG: (**a**) Annular channel; (**b**) Radial channel.

**Figure 5:**
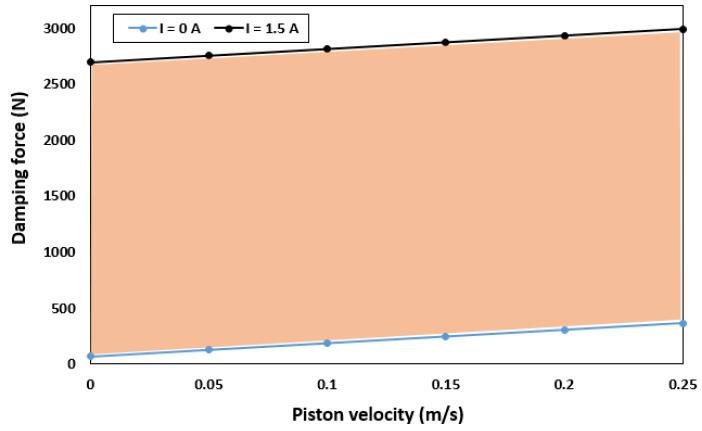
Variation of damping force of the HARMRD with piston velocity for initial design values.

Unlike the magnetic field from the magnets, that from the coil, passes through both radial and annular directions and extends to the core cylinder to form a complete cycle, as shown in Figure 3a. After supplying a current of 1.5 A to the coil, the magnetic field densities generated for both radial and annular gaps were 0.56 T and 0.44 T, respectively. A typical magnetic field problem is described by defining its geometry, material properties, magnetic input, and boundary conditions. Based on the properties of MR fluid (MRF-132DG) [25] and FEM analysis results for electromagnetic characteristics shown in Figure 3, relationship between yield stress and magnetic field intensity can be obtained as presented in Figure 4. Figure 4a,b depict the relationship between magnetic flux density (B) and yield stress $\left(\tau_{\gamma }\right)$ in the annular and radial channels, respectively. The data points in Figure 4 were fitted into a polynomial curve using the least square method in MATLAB simulation software. Evidently, the radial channel generates a higher yield stress and magnetic field than the annular duct. The yield stresses in the two channels were deduced from Figure 4a,b as follows.

(16) $$\tau_{ya}=\left(-1.2324\times B_{a}^{3}+1.4074\times B_{a}^{2}+0.1604\times Ba+0.0005\right)\times 10^{5},$$

(17) $$\tau_{yr}=\left(-1.1602\times B_{r}^{3}+1.3716\times B_{r}^{2}+0.1619\times Br+0.0010\right)\times 10^{5}$$

where $\tau_{ya}$ and $\tau_{yr}$ are the yield stresses in the annular and radial channels, respectively. To evaluate the performance of the proposed model, the damping force was calculated based on the equations in Section 3. Figure 5 shows the minimum damping force when no current is supplied to the coil (field-off condition) and the maximum damping force generated when the coil is excited (field-on condition). When the field is off (I = 0 A), a damping force of 70.507–365.98 N is generated for piston velocities of 0 and 0.25 m/s, respectively. By contrast, when the field is on (I = 1.5 A), the damping force increases from 2696.3 N to 2991.8 N for the same piston velocities. In addition, the highest dynamic range of 3.19 and 47.63 was obtained at a piston velocity of 0.05 m/s for field-off and field-on states, respectively. However, as the piston velocity increases, the dynamic range decreases significantly. For a constant speed of 0.25 m/s, dynamic ranges of 2.24 and 11.13 were obtained for the field-off and field-on states, respectively.

## 5. Optimization of the HARMR Valve

**Figure 6:**
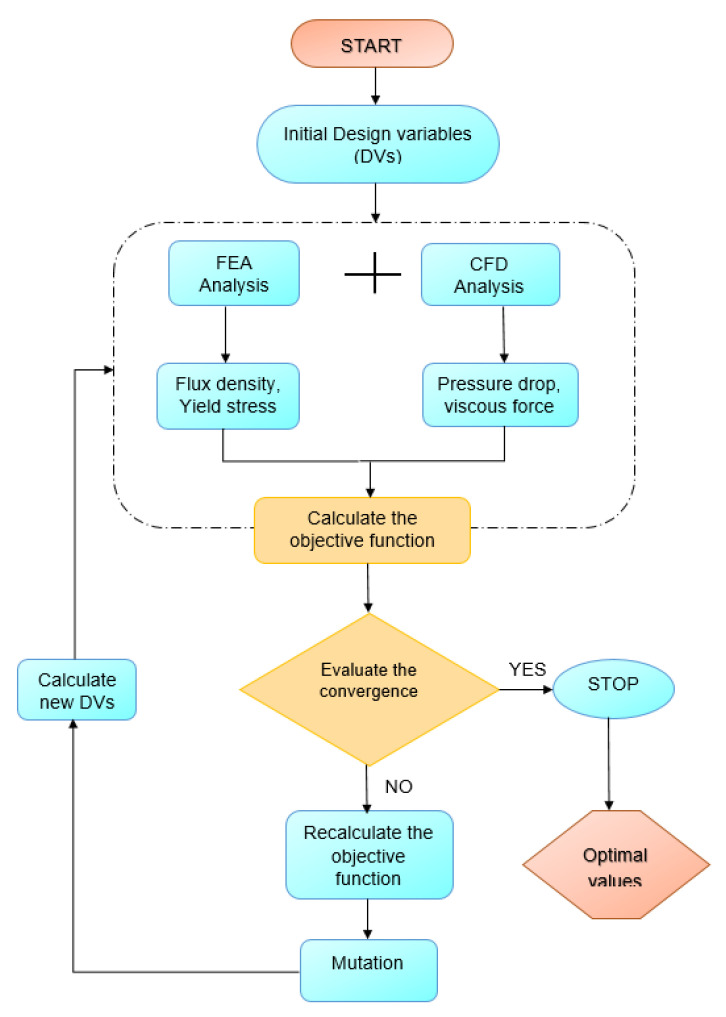
Flowchart of genetic algorithm used for optimization.

After obtaining the performance indices from the initial design parameters, a multi-objective genetic algorithm (MGA) is used to optimize the HARMRD. MGA is based on concepts from natural selection and genetics. It is commonly used to obtain high-quality solutions to search and optimization problems. The optimization process used in this study is depicted in Figure 6. During optimization, seven design variables (DVs) were selected based on their effects on the performance indices, including the damping force and dynamic range. The selected DVs are the piston radius $\left(R_{3}\right)$, radius of circular disc $\left(R_{2}\right)$, radius of the core from the central hole $\left(R_{1}\right)$, coil width (W), piston radius $\left(R_{pr}\right)$, annular duct $\left(t_{g}\right)$, and main pole (b). The upper and lower bounds of the design variables are listed in Table 2. In this study, the damping force and dynamic range were selected as the fitness functions. Ride comfort and suspension travel are two important factors in vehicle suspension design. To maintain the suspension travel short, a larger damping force is required, whereas to achieve better ride comfort, a low damping force and larger dynamic range are required. The fitness function is expressed as follows [26]:

(18) $$OBJ=\alpha \frac{F_{D}{}^{*}}{F_{D}}+\beta \frac{D_{r}{}^{*}}{D_{r}},$$

where $F_{D}{}^{*}$, $D_{r}{}^{*}$ are the reference damping force and reference dynamic range, respectively, and α,  and β are the weighting factors. In addition, the summation of both weighting factors should be equal to 1. To balance both damping force and dynamic range, the weighting factors were chosen as 0.5 in this work.

**Figure 7:**
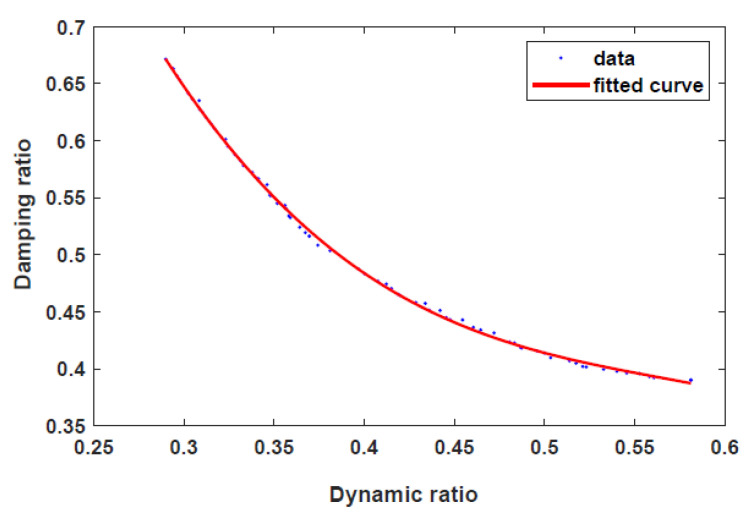
Pareto front between objective functions.

**Figure 8:**
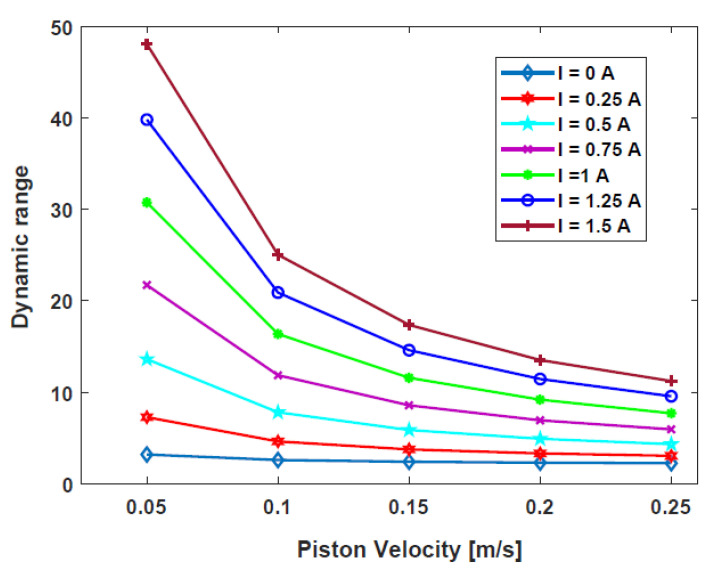
Dynamic ranges of the HARMRD under various excitation velocities.

Figure 7 shows the Pareto front generated between the fitness functions. Evidently, the dynamic range decreases with increasing damping force. The optimal design parameters are listed in Table 3. Figure 8 shows the dynamic ranges of the optimal HARMRD under various velocity excitations. As it is apparent from this figure, the dynamic range decreases as the piston velocity increases. The wide range of controllability is confirmed by the highest dynamic range experienced at the excitation current of I = 1.5 A. Moreover, the dynamic range obtained during the off-field state indicates that the proposed model addresses the fail-safe problem. The relationship between the dynamic range and piston velocity for the optimal model was determined using MATLAB/Simulink under current excitations ranging from 0 to 1.5 A. During the simulation, a sinusoidal displacement with an amplitude (A) of 0.075 m and frequency of 2 Hz was used.

**Figure 9:**
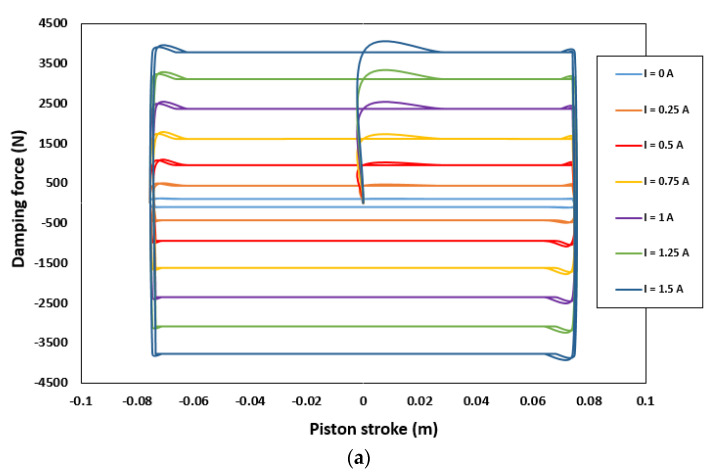

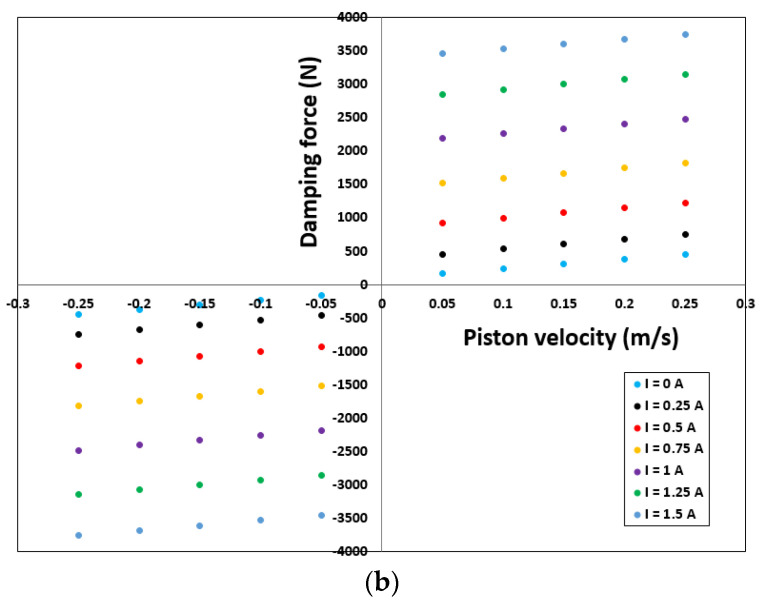
Damping force characteristics: (**a**) damping force versus piston stroke; (**b**) damping force versus piston velocity.

**Figure 10:**
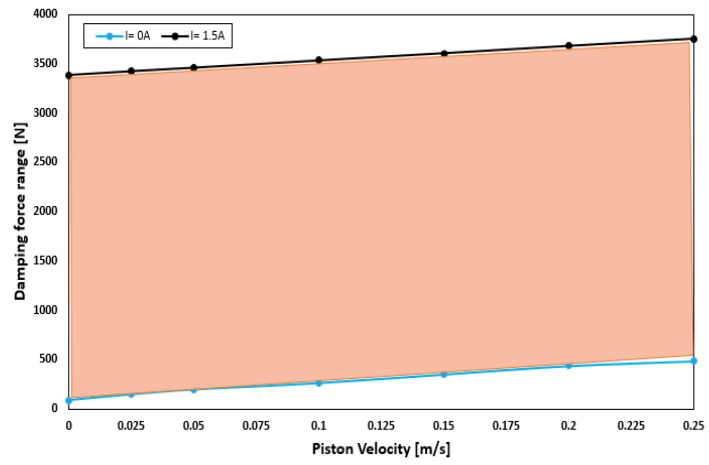
Variation of damping force of the HARMRD with piston velocity with optimized parameter.

The force-piston stroke and force-piston velocity are shown in Figure 9a,b, respectively. Figure 9a depicts the relationship between the damping force and piston stroke (FD-S) for various current excitations. Evidently, FD-S curves gradually rise with the current supplied. Moreover, the damping forces for the compression and extension strokes are symmetric. The small deformation around the top-left corner and bottom-right corner is due to insufficient gas pressure in the accumulator. As shown in Figure 9a, a minimum damping force of 445.7 N was generated in the off-field state (I = 0 A). By contrast, a maximum damping force of 3753.9 N was obtained when the maximum current (I = 1.5 A) was applied. This indicates that the optimal HARMR model is capable of generating a wide range of damping force (445.7–3753.9 N), which is necessary for achieving better driving comfort. Figure 9b shows the variation of damping force with piston velocity under various current inputs. It is evident that the proposed HARMR model can mimic the behavior of an MR damper. Generally, it is difficult to replicate the MR property in an off-field state. In addition, the damping force increased as the excitation and piston velocities increased. The proposed model generates a damping force range of 445.7–3753.9 N under the piston velocity of 0.25 m/s. Figure 10 illustrates the damping force range under various velocity inputs. The wide damping force range enables the use of short stroke which is essential for improving the ride comfort and vehicle handling. When the field is off (I = 0 A), a damping force of 87.25–445.7 N is generated for piston velocities of 0 and 0.25 m/s, respectively. By contrast, when the field is on (I = 1.5 A), the damping force increases from 3386.4 N to 3753.9 N for the same piston velocities. Compared with the results in Figure 5, it can be clearly seen that the damping force range increased after optimization. After optimization, the damping force is 3753.9 N, which is 20% higher than that of 2991.8 N before optimization, and the suspension travel can be restricted with sufficient damping force. The dynamic range under the condition of piston velocity of 0.05 m/s is evaluated by 3.19 and 47.63 at field-off and field-on, respectively, before optimization. However, it increased to values of 3.19 and 48.08 under the same conditions after optimization. These results mean that the damping force can be greatly improved without reducing the dynamic range through optimization. A typical passive damper has a damping force of about 130 to 400 N at a piston velocity of 0.025 to 0.2 m/s [27]. In the case of the model proposed in this paper, it was confirmed to have a damping force of 147 to 437 N for the same piston speed in the field-off state. From these results, it is confirmed that the proposed model has adequate performance for fail-safe issues. When the damping force per unit volume is calculated based on the maximum damping force, it has values of 0.0535 N/mm^3^, 0.0388 N/mm^3^, and 0.0597 N/mm^3^ for Bai’s model, RD-1005-3 model of LORD Corp. (Cary, NC, USA), and the model proposed in this paper, respectively. The maximum damping force under the same current input of 1.5 A and the piston speed of 0.19 m/s is considered. The volume is calculated using the piston radius, piston rod radius and stroke. From this, it is confirmed that improved damping force can be obtained by using the proposed model compared with both model in previous research work and commercial model.

## 6. Performance Evaluation of the HARMRD

**Figure 11:**
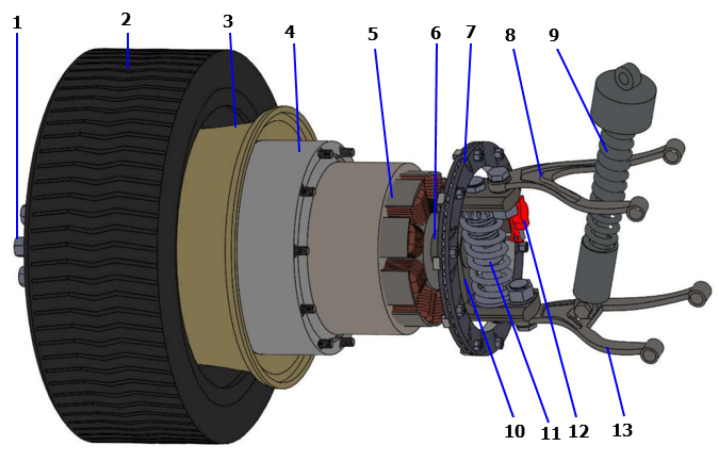
Physical structure of the HARMRD for electrical IWM suspension. 1. Nuts, 2. Tire, 3. Wheel, 4. Motor rotor, 5. Motor stator, 6. Hub shaft, 7. Brake disk, 8. Upper control arm, 9. HARMRD, 10. Hub, 11. DAV damper, 12. Brake caliper, 13. Lower control arm.

EVs are typically powered by either a centralized motor or an IWM, depending on the vehicle design. Motors arrangement is the primary difference between these two EV designs. In the IWM design, a motor is installed on each wheel to directly supply torque to the wheels. By contrast, in a conventional layout, the motor is centralized, and the torque is transferred to the wheels via the drive shaft. The IWM configuration offers advantages over conventional models, such as improved efficiency, easy generation of forward and reverse torques, and improved performance of systems such as the anti-lock brake system and traction control system [28]. However, driving comfort and handling stability are primary considerations in the design of the suspension system. In previous studies, the IWMs integrated into the wheels increased the unsprung mass, which resulted in the deterioration of the tire dynamic road and body vibration acceleration. Several methods have been proposed to tackle the weight issue, including the in-wheel switched reluctant motor (IWSRM) and permanent magnetic synchronous motor (PMSM) [29]. However, both the IWSRM and PMSM models have been found to experience radial force imbalance due to rotor eccentricity, which affects the ride comfort. However, an IWSRM is affordable and simple. In this study, an SRM coupled inside a wheel was modeled, and a dynamic vibration absorbing structure was connected to the unsprung mass to suppress the vibration of the unsprung mass. Figure 11 shows the proposed EV suspension powered by the IWM. The proposed IWM comprises 13 components, which are grouped into three parts (wheel and tire, SRM, and suspension dampers). To suppress motor vibration, a small passive damper was installed inside the stator cover, which was connected to the hub shaft. In addition to the small damper in the DVA structure, HARMRD was installed between the lower and upper arms of the suspension to control road-induced vibration.

**Figure 12:**
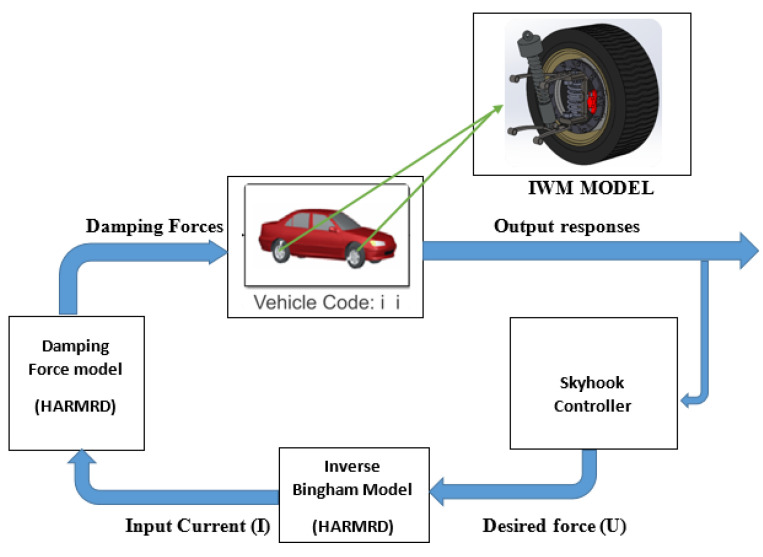
Block diagram of co-simulation of EV suspension.

A co-simulation software (CarSim, V 9.0, Mechanical Simululation Corporation, Ann Arbor, MI, USA) was used to evaluate the effectiveness of the proposed HARMR model for an EV powered by an IWM. The CarSim interface is divided into three main parts: vehicle parameters and drive cycle, MATLAB/Simulink interface, and simulated results. CarSim is widely used to simulate the vehicle performance in terms of ride comfort, handling stability, and power. A D-class sedan with independent suspensions was used in the simulation. Figure 12 illustrates the simulation in MATLAB/Simulink in conjunction with CarSim. The damping forces (*F_D_*) supplied on each tire were considered as an external input to CarSim, and they were controlled by the Skyhook controller. In CarSim, the full vehicle parameters are specified based on a real vehicle. The considered parameters include the sprung mass, unsprung mass, wheelbase, vehicle width, and vehicle height. The parameters for the full vehicle are listed in Table 4. Five outputs were obtained from the sensor, namely force rate, body velocity, body deflection, tire deformation, and vertical acceleration. During the simulation, bump and random input roads were adopted from the CarSim software. The moving vehicle velocity for the bump and random road was fixed to 60 km/h. Here, a normal vehicle powered by an engine and an EV powered by an IWM were compared under the same road conditions. For simplicity, only the front-left suspension responses were considered for the full vehicle. For the riding test, a bump road input with amplitudes of 35 mm and 400 mm was used. A vehicle velocity of 60 km/h was used.

**Figure 13:**
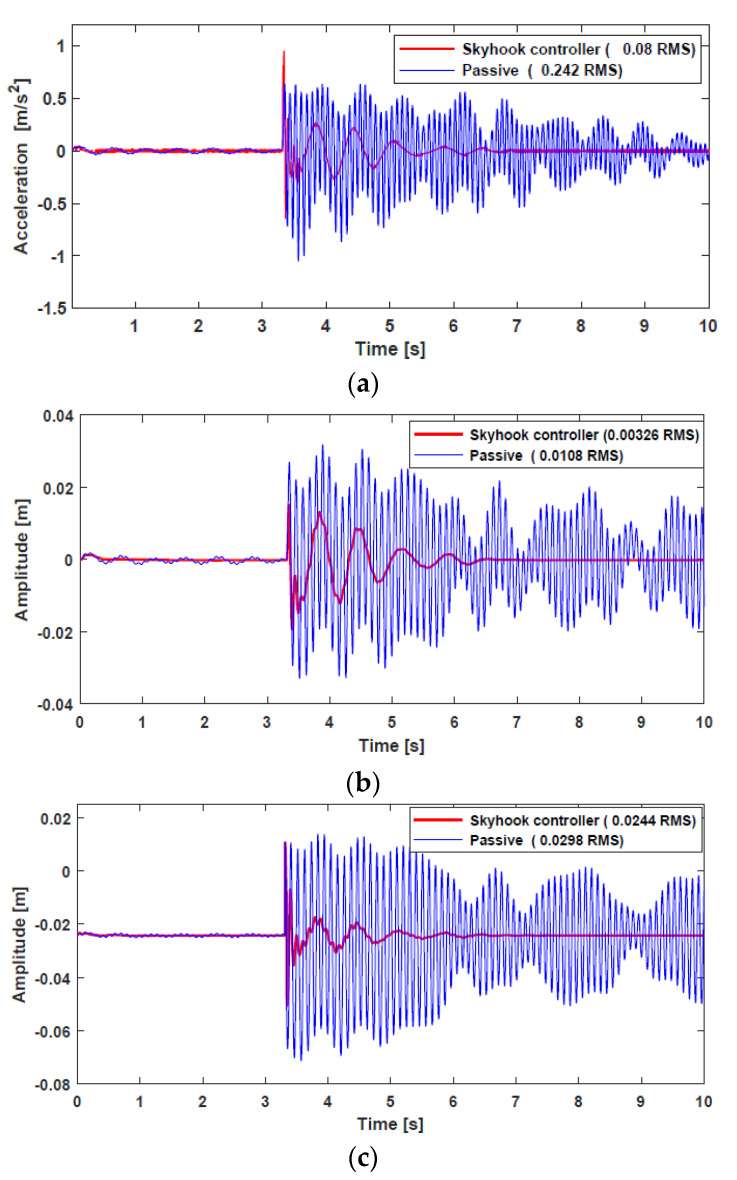
Time responses for a normal vehicle under bump road excitation: (**a**) vertical acceleration from the center of gravity; (**b**) suspension deflection; (**c**) tire deflection.

**Figure 14:**
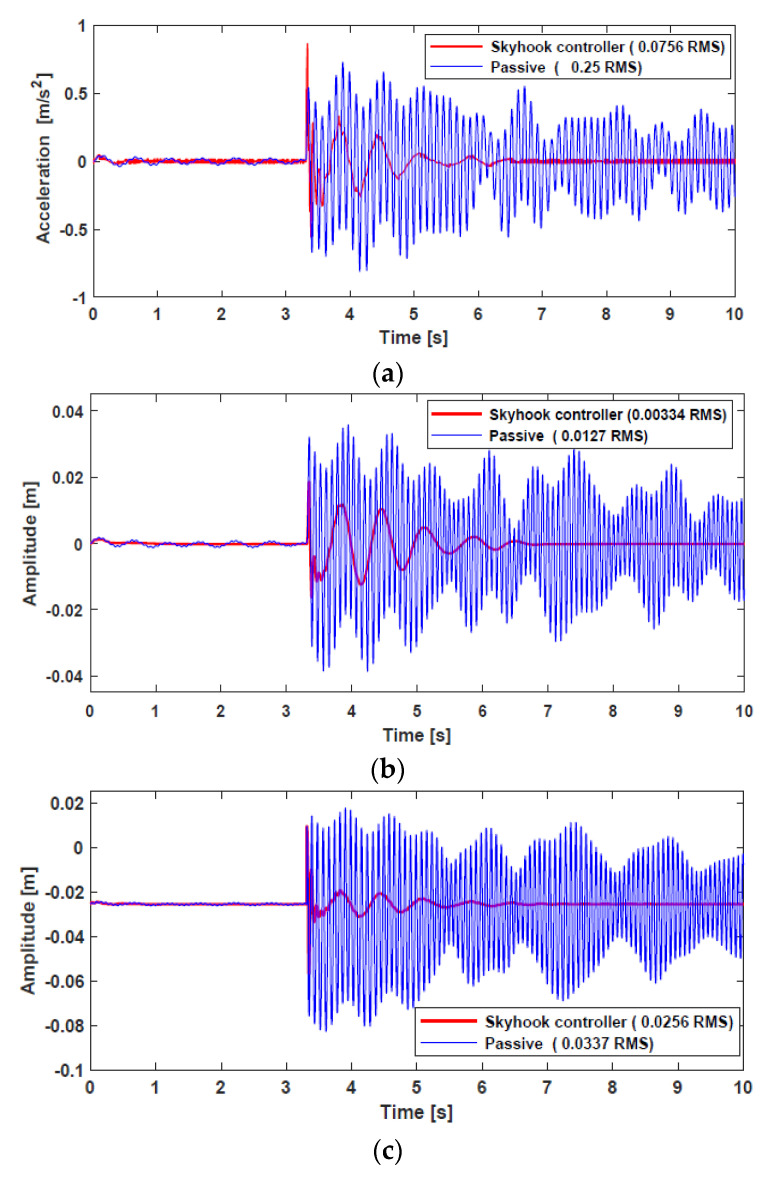
Time responses for IWM under bump road excitation: (**a**) vertical acceleration from the center of gravity; (**b**) suspension deflection; (**c**) tire deflection.

The vertical acceleration, suspension deflection, and tire deflection responses of a normal vehicle are shown in Figure 13. Two cases were investigated and compared during the simulation. First, the vehicle in CarSim was simulated without an external force, that is, passive suspension. Second, the external force from the HARMRD, controlled by the skyhook controller, was supplied to the vehicle. It is evident that the vehicle controlled by the proposed damper suppressed undesirable vibrations compared with the passive suspension. In addition, the proposed model exhibited a fast settling time after the vehicle hit the bump, which confirms the improvement in ride comfort achieved by the proposed model. To statistically verify the performance, the root mean square (RMS) for both the passive and controlled responses was calculated. The RMS values for passive responses decreased by 66.9%, 69.18%, and 18.12% for vertical acceleration, body deflection, and tire deflection, respectively. Figure 14 shows the time responses for a vehicle powered by IWM. Evidently, the proposed HARMD attenuates the vibrations for acceleration, suspension deflection, and tire deflection. The difference in responses between a vehicle powered by an engine and another powered by IWMs was insignificant. However, the RMS values for the IWM slightly increased compared with the RMS values for a normal vehicle. The passive RMS values for vertical acceleration, suspension deflection, and tire deflection reduced by 69.76%, 73.7%, and 24.03%, respectively.

According to ISO 2631-1, the ride comfort can be mathematically identified and quantified based on the relative discomfort associated with vehicle motion. The frequency weighted RMS Acceleration calculates how intensive mechanical vibrations affect the human body in vehicles. To quantify the relative discomfort, the frequency weighted RMS acceleration for vertical accelerations was calculated as follows.

(19) AW=[1T∫0TA(w)2]12

where A(w) is the frequency weighted acceleration and T is the exposure time. According to ISO 2631-1, when the frequency weighted RMS acceleration is lower than 0.32, the Discomfort level is “Not uncomfortable”, and when it is greater than 0.32 and less than 0.5, the Discomfort level is “A little uncomfortable”. In the case of a normal vehicle, when the passive damper is used, the frequency weighted RMS acceleration value is 0.42, and when the proposed model is applied, it decreases to a value of 0.18, which satisfies the lowest Discomfort level. Even in the case of the IWM vehicle, when the passive damper is used, the frequency weighted RMS acceleration value is 0.46, and when the proposed model is applied, it can be confirmed that the lowest discomfort level is satisfied by decreasing to a value of 0.16.

**Figure 15:**
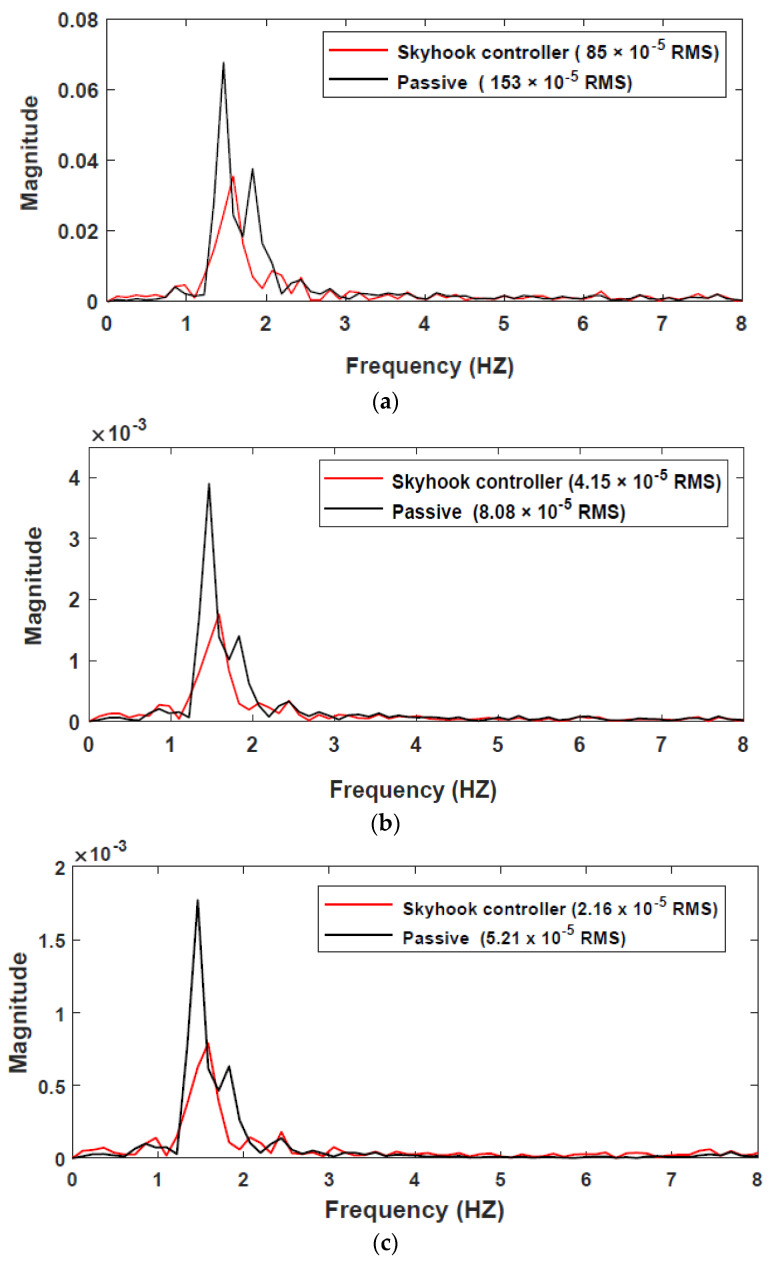
Frequency responses for IWM under random road excitation: (**a**) vertical acceleration from the center of gravity; (**b**) suspension deflection; (**c**) tire deflection.

**Figure 16:**
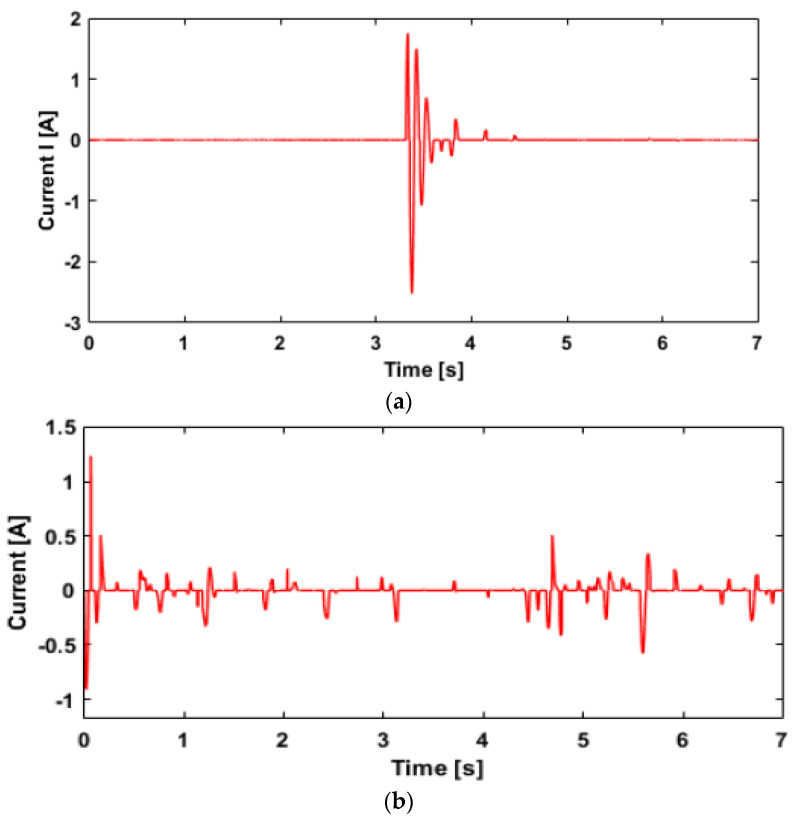
Current input: (**a**) bump road input; (**b**) random road input.

In addition, the proposed model was subjected to a random road to test its capability of attenuating random vibrations. Different grades of a random road are used depending on the purpose and type of vehicles. In this study, a grade B random road was selected as the testing road in CarSim, which can be described as follow [31]

(20) $$\dot{x}\left(t\right)=-2\pi f_{n}vx\left(t\right)+2\pi f_{0}\sqrt{s_{h}\left(f_{0}\right)vw\left(t\right)},$$

where $\dot{x}\left(t\right)\;$ represents the road profile, $f_{n}=0.01$ is the cut-off frequency, $f_{0}=0.1$ is the spatial frequency, v is the assigned vehicle velocity selected as 60, $s_{h}\left(f_{0}\right)$ is the road roughness coefficient, and w(t) is the random white noise. During the simulation, a skyhook controller was used to control the input damping force of each tire. The time responses from the random road are not clear to interpret because of the noise; therefore, the frequency responses obtained from the time domain were used to compare controlled and passive responses. Figure 15 shows the frequency responses of a vehicle powered by the IWM. The simulation results indicated that the controlled responses outperformed the passive responses in terms of dissipating energy generated from road unevenness. In addition, all the first peak resonances were in the vicinity of frequencies (1–2 Hz). According to ISO 2631 [32], maximum sensitive vibration to the human body ranges from (4–8 Hz). The RMS values of the body vertical acceleration, suspension deflection, and tire deflection confirm that the proposed damper model has a significant impact on lowering the RMS values compared with the passive values. The power supplied to the semi-active HARMR damper for both bumpy and random road input is illustrated in Figure 16a,b, respectively. Evidently, the proposed model is efficiently able to suppress the vibration for both bumpy and random roads with the generated current of about 1.7 A and 1.2 A, respectively.

## 7. Conclusions

In this study, HARMRD was designed and modeled to improve the ride comfort of EVs. The simulation results indicated that HARMRD generates a higher damping force than the conventional MR damper and provides a wider range of vibration controllability. To demonstrate these benefits in terms of the vehicle suspension system, the overall performance of the HARMRD on ride comfort was evaluated via co-simulation using virtual full-vehicle dynamics in CarSim and MATLAB/Simulink. In the simulation, a simple but highly effective skyhook controller was used to control the current supplied to the HARMRD. The simulation results for random road inputs confirmed that the controlled proposed model improved both ride comfort and road holding by significantly suppressing unwanted vibrations compared with passive suspension. It is finally remarked that an experimental validation of the proposed HARMRD needs to be conducted in the near future through a hardware-in-the-loop simulation and real road test.

## Figures and Tables

**Table 1:** Structural dimensions of the proposed HARMRD.

Parameter	Symbol	Value
Piston rod radius	$R_{rp}$	5 [mm]
Piston radius	$R_{3}$	20 [mm]
Annular duct	$t_{g}$	1 [mm]
Radial duct	$t_{r}$	1 [mm]
Flux return	$t_{c}$	5 [mm]
Central hole radius	$R_{o}$	3 [mm]
Magnetic pole radius	$R_{2}$	14 [mm]
Height of magnet	$H_{m}$	3 [mm]
Height of core	$L_{c}$	26 [mm]
Core radius	$R_{1}$	12 [mm]
Minor pole	a	2 [mm]
Big pole	b	5 [mm]
Piston valve length	L	48 [mm]
Coil turns	N	304 turns

**Table 2:** Lower and upper bounds of design variables.

Parameter	Lower Bound	Upper Bound
$\mathrm{Piston}\;\mathrm{rod}\;\mathrm{radius}\;\left(R_{rp}\right)$	5 mm	7 mm
$\mathrm{Piston}\;\mathrm{radius}\;\left(R_{3}\right)$	20 mm	23 mm
$\mathrm{Annular}\;\mathrm{duct}\;\left(t_{g}\right)$	0.8 mm	1 mm
$\mathrm{Magnetic}\;\mathrm{pole}\;\mathrm{radius}\;\left(R_{2}\right)$	14 mm	16 mm
$\mathrm{Core}\;\mathrm{radius}\;\left(R_{1}\right)$	12 mm	14 mm
Big pole (b)	5 mm	5 mm

**Table 3:** Optimal geometric parameters for the HARMRD.

SN	Parameters	Dimensions
1	$\mathrm{Annular}\;\mathrm{duct}\;\left(t_{g}\right)$	0.97 mm
2	$\mathrm{Piston}\;\mathrm{radius}\;\left(R_{3}\right)$	20.7 mm
3	$\mathrm{Piston}\;\mathrm{road}\;\mathrm{radius}\;\left(R_{rp}\right)$	6 mm
4	$\mathrm{Magnetic}\;\mathrm{pole}\;\mathrm{radius}\;\left(R_{2}\right)$	16 mm
5	Big pole (b)	6 mm
6	$\mathrm{Core}\;\mathrm{radius}\;\left(R_{1}\right)$	13 mm
7	Damping force	3753.9 N
8	Total length (L)	48 mm

**Table 4:** Passenger vehicle parameters [30].

Parameter	Value	Parameter	Value
Sprung mass	1370 kg	Unprung mass (normal vehicle)	80 kg
Longitudinal inertia moment	$356.3\;\mathrm{kg}\cdot m^{2}$	Unsprung mass (IWM)	$140\;\mathrm{kg}\cdot m^{2}$
Front suspension stiffness	153 kN/m	rear suspension stiffness	82 kN/m
Tire stiffness constant	230 kN/m	Damper coefficient	1534 N·s/m
Distance from C.G to the front suspension	1.11 m	Distance from C.G to the rear suspension	1.166 m
Distance from C.G to left suspension	0.335 m	Distance from C.G to right suspension	0.335 m

## Data Availability

Not applicable.
